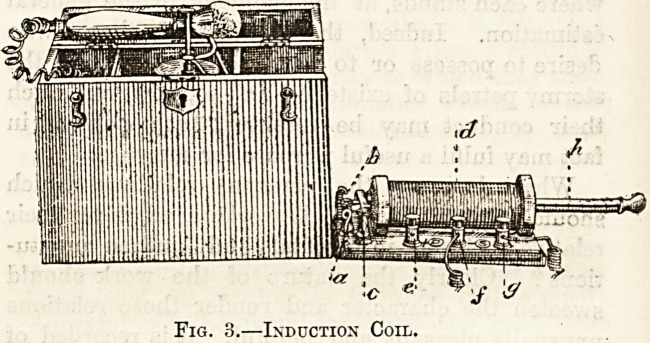# The Hospital. Nursing Section

**Published:** 1905-07-29

**Authors:** 


### The Hospital.

### Nursing Section.

Contributions for this Section of "The Hospital" should be addressed to the Editor, "The Hospital" Nursing Section, 28 & 29 Southampton Street, Strand, London, W.C.

No. 983.—Vol. XXXVIII. SATURDAY, JULY 29, 1905.

### Notes on News from the Nursing World.

### THE QUEEN AND MISS PETER.

A great and well-deserved honour was paid by the Queen to Miss Peter. On Saturday the retiring general superintendent of Queen Victoria's Jubilee Institute was received at Buckingham Palace by her Majesty. The Queen having, in gracious language, expressed to Miss Peter her regret at the loss which the Institute would sustain by her resignation after seventeen years of devoted service, presented her with a brooch. Her Majesty also sent, through the general superintendent, a special message of encouragement in their work to the 1,400 Queen's nurses who are tending the sick poor in their homes throughout England, Scotland, Ireland, and Wales.

### THE QUEEN AT A CHELSEA HOSPITAL.

There was considerable excitement amongst the nurses of the Victoria Hospital for Children at Chelsea when it became known that the Queen had announced her intention of paying a surprise visit on Saturday afternoon. The notice given was very short, but the children had been given such few directions about their behaviour as were deemed necessary, more particularly the little boy patient to whom fell the honour of presenting the Queen with a bouquet of La France roses. He had been especially told not to forget his bow, and had so taken the instructions to heart that when the auspicious moment came he murmured to the Queen as he handed the flowers, "Bow." Her Majesty was much amused when the meaning of the strange monosyllable addressed to her was explained. She afterwards took out one of her flowers and gave it to a little girl suffering from St. Vitus's Dance, who was shut off from the others by a screen. Her Majesty, who spent an hour in the wards, said that she thought the children looked very happy, and that she was very pleased with the hospital. The staff in their turn were much gratified by the readiness with which the Queen gave her consent to the proposal that the last ward to be finished shall bear the name of "Queen Alexandra Ward" and intimated her intention of opening it when it is ready for occupation.

### JAPANESE HONOURS FOR A DEVOTED ENGLISHWOMAN.

Mrs. Richardson, whose acceptance and wearing of the Sixth Order of the Crown, conferred upon her by the Mikado of Japan, has been sanctioned by the King, is now on her way to England. She left this country early last year to work with the Japanese Red Cross Society at Tokio, and is the only English nurse who has enjoyed that privilege. Her services have been so much appreciated in Japan that she has received, with the consent of the Mikado, the Order of Merit from the Red Cross Society, and in addition to the two orders, two medals have been given to her. Mrs. Richardson, who also holds the South African medal, worked for some time in the military hospitals in South Africa.

### REPORT OF THE SELECT COMMITTEE ON REGISTRATION.

We are informed that the Select Committee on the Registration of Nurses completed their inquiry on Tuesday, and that the report was laid the same night on the table of the House of Commons.

### MENTAL NURSES AND STATE REGISTRATION.

In his speech last week at the annual meeting of the Medico-Psychological Association Dr. J. Outterson Wood, the new president, discussed the question of nursing. We report this portion of his speech in another column. Dr. Savage at the annual dinner said the association had now become so powerful that he believed in the event of State registration being proposed by the Government they would be bound to recognise it and defer to its opinion in respect to any changes which might be in contemplation. The present Government, we have the best authority for affirming, have not the least intention of proposing State registration for nurses, but we gladly record our opinion that in any scheme of this character which may hereafter be considered by Parliament the interests of asylum workers could not possibly be overlooked. If most of them are still not fully-trained hospital nurses a large number are, and in the future this will increasingly be the case. They are therefore fully entitled to have a voice in a matter which, whenever it is seriously broached in Parliament, must more or less directly affect all branches of nursing.

### THE RULES AT HORTON ASYLUM.

At the trial of Walter Clapham for the murder of his wife, who was an inmate of Horton Asylum, two facts of special interest and importance transpired in evidence. One was that Clapham, who was subsequently declared to be insane himself, killed his wife by cutting her throat with a razor in a bedroom while the nurse was absent. It appears that there is a rule at the institution that the nurses are not to go too near the visitors, so as not to interfere with their private conversation. In this case the nurse affirmed that though she left the room she did not go out of hearing, the door of the room being open. As to this we can only remark that the rules should be stringent enough to render it impossible for a patient to be killed by a visitor in the absence of a nurse or *vice versa*. As to the other point, counsel for Clapham rested his defence mainly on the ground that his client's mind had been gradually unhinged by the receipt of letters from his wife, who wrote him frequently to bring her means to put an end to her life. There was no denial that these letters were written and sent out from the asylum. The rules of the institution must have been transgressed, and we are glad to learn from the medical superintendent that the matter is now under review by the committee.

### RUNNING A NURSES' HOME.

Some interesting statements were made at the Halifax County Court last week by Miss A. W. Paul, of the "Nurses' Home," 4 Savile Terrace, on her examination by the Deputy Official Receiver. The liabilities amounted to £45 11s. 4d. and the assets to £9 9s. 2d. The debtor said that she commenced the home in 1890 with a capital of only a few pounds, having bought it and the furniture for £179. She had an overdraft on the Halifax Bank, and an account with a Manchester Bank. She was "practically clear of debt" when she opened the home. Her business, she continued, was to supply nurses, when and where required. She drew their fees, paid them, and kept them in the home when not employed. At first she had about 11 nurses, and her average number for some time since was eight. She charged for the nurses 25s. per week for non-infectious cases, and a guinea and a-half for infectious cases. To the nurses, she paid £34 a year and she gave them three weeks' holiday. She attributed her failure chiefly to the opening of a rival establishment by a nurse who was formerly with her, though the exceedingly low death-rate of Halifax was a contributing element. The examination was closed, but the experience of Miss Paul will, we hope, have the salutary effect of discouraging attempts to run nurses' homes under similar conditions.

### THE FRICTION AT LIMERICK COUNTY INFIRMARY.

Miss Mayne, matron of the Limerick County Infirmary, having successfully cleared her reputation in a court of law from the aspersions which had been cast upon it, has since had to run the gauntlet of pressure on the part of the governors of the institution to answer a question which the Lord Chief Justice assured her at the trial she need not answer. However in the result, they were content to pass a resolution that the remaining servant of the four who were in the kitchen on the morning that iodoform was thrown on Mrs. O'Brien's clothes should be asked to resign, and to abandon the idea of trying to obtain the name of the actual offender. On the subject of uniform an Irish magistrate sends us a letter, which we publish to-day with pleasure, though it does not alter our opinion as to Mrs. O'Brien's suggestion that the nurses should attend a ball in the uniform of the Limerick Infirmary.

### GUARDIANS AND THE LOCAL GOVERNMENT BOARD.

At the last meeting of the Carmarthen Guardians a letter from the Local Government Board insisting upon the appointment of a trained nurse in place of the untrained nurse recently selected by the Guardians, was discussed, and a curious decision was arrived at. The Guardians determined to make a second appeal to the Local Government Board to confirm the appointment already made, the only ground for the appeal being that the nurse they have chosen has had experience at the Joint Counties Asylum. In these circumstances, we cannot imagine that the Local Government Board will give way. The experience at a mental asylum of the nurse appointed by the Guardians does not constitute training, and there is no reason why an exception should be made in this instance.

### SALARIES AT STOKE WORKHOUSE INFIRMARY.

The Stoke-upon-Trent guardians have very wisely decided to consider the general question of nursing at the Workhouse, and to reconsider the recommendation of the Finance Committee to increase the salaries of certain nurses. This decision is due to the fact that having very properly instituted inquiries in other Unions as to the salaries paid, the Stoke guardians have ascertained that the nurses in their own employ are not adequately remunerated for their services.

### QUEEN ALEXANDRA'S MILITARY NURSING SERVICE.

Three new staff nurses have been appointed to Queen Alexandra's Imperial Military Nursing Service—namely, Miss M. Plaskitt, Miss F. M. Tosh, and Miss C. M. Williams. Staff Nurse E. C. Ellis has been transferred to Cambridge Hospital, Aldershot; Staff Nurse H. L. A. Jack, from Princess Louise Hospital, Alton, to the Royal Hospital, Woolwich; Staff Nurse M. C. Johnston to the Military Hospital, Devonport; and Staff Nurse F. M. Tosh to the Princess Louise Hospital, Alton. Sisters A. R. F. Auchmuty, E. M. Denne, K. Pearse, and Staff Nurse A. M. Pagan are held in readiness for service abroad. Sisters E. J. Martin, J. Hoadley, R.R.C., A. Nixon, S. I. Snowdon, M. E. Harding, L. M. Todd, M. Steenson, and Staff Nurse A. B. Cameron are due to return from South Africa during the forthcoming trooping season; and the appointments of Miss K. A. Allsop, Miss H. L. A. Jack, and Miss G. S. Jacob as staff nurses have been confirmed.

### QUEEN'S NURSES AND HOSPITAL SUNDAY FUND.

Last week a deputation from Queen Victoria's Jubilee Institute waited upon the General Purposes Committee of the Metropolitan Hospital Sunday Fund for the purpose of advocating the claims of the Institute to share in the benefits of the Fund. It was urged that the great work done by the Queen's nurses in the metropolis in relieving pressure at the hospital by attending thousands of the sick poor in their own homes entitled the Institute to recognition, and it was pointed out that in several of the great provincial cities the Hospital Sunday and Saturday Funds allocated money to the Queen's nurses. In reply, the Committee stated that the constitution of the Fund does not allow of grants being made to bodies other than actual hospitals and dispensaries in the metropolis, but held out hopes that modifications might be considered in the near future, without varying the existing principles of the Fund in making grants within the metropolitan area.

### THE NEW MATRON OF BLACKPOOL ISOLATION HOSPITAL.

The authorities of isolation hospitals are frequently far from careful in the choice of matrons for these important institutions. We, therefore, welcome the selection of a Nightingale nurse as matron of Blackpool Infectious Diseases Hospital. Miss Procter, whose appointment we announce to-day, was trained at the Nightingale School, St. Thomas's Hospital, and the Cardiff Infectious Diseases Hospital, of which she has been deputy-matron. She has also been matron of two other Welsh isolation hospitals, and her training and career justify the belief that she will prove equal to her new duties at Blackpool.

### MASSAGE FOR SOLDIERS.

In the Chancery Division on Tuesday an application was made by the Incorporated Society of Trained Masseuses for the sanction of the Court to an alteration in the Society's memorandum and articles of association so as to enable it to include in its training and examinations men as well as women. The society, which was formed in 1900, has recently been applied to by the War Office asking for the benefit of lectures and examinations to the members of the Royal Army Medical Corps. They proposed that the lectures should be given to men, though not to allow them to become members of the association. Mr. Justice Swinfen Eady approved the addition of the necessary words in three places in the articles of association.

### STRIKING SUCCESS AT KINGSWOOD.

At the annual meeting of the Kingswood District Nursing Association, Mrs. C. E. Hobhouse drew attention to the remarkable progress made by this organisation. She reminded the audience—which included many well-known people—that three years ago the home was started with one superintendent and one probationer; whereas now there is a working staff of 12. During the three years more than 500 patients had been attended each year, at a cost to the neighbourhood, in money subscribed and donations, of £270 a year. They had moved from the original small home into a larger one, and this was now too small. They had also been able to form a branch at Hanham, and their school had been worked so well that the Central Midwives Board had recognised them as a training-place. The home had never been in debt, and the expenditure last year was a trifle less than the income. The position quite justifies the congratulations which were offered to the committee by Lady Lucy Hicks-Beach, who, addressing the meeting in her capacity of a member of the Executive Committee of the Gloucester County Nursing Association, said that the institution at Kingswood was of general value and deserved the warm support of all living in the neighbourhood.

### THE RETIRING MATRON OF PORTSMOUTH PARISH INFIRMARY.

We understand that Miss A. B. Clarke, who was recently appointed matron of Leicester Poor-law Infirmary, having returned to her post at Portsmouth Parish Infirmary for a time before taking up her new duties, the patients and staff have presented her with mementoes of their appreciation of her work, the gift of the nurses being a silver tea service. Miss Clarke, in acknowledging the latter, thanked the nurses for the bright and willing manner in which they have obeyed and carried out her wishes and instructions at all times. She also asked them to be as loyal and kind to the new matron as they had been to her, and she impressed upon them the importance of doing their best, not only to alleviate the pain and sufferings of the patients, but likewise to relieve the monotony of the lives of those who have no actual pain.

### LACK OF NURSES IN THE NORTH-WEST OF AMERICA.

While the supply of nurses in some of the great cities in the United States is in excess of the demand, the smaller but prosperous towns in the North-West are calling out for more. Medical men in Idaho Falls, Idaho, are said to be in despair, and at Wessington Springs, South Dakota, a nurse is badly wanted. The scale of payment is as good as in the cities, and there is no competition. It is notable that of the 250 nurses who, it is estimated, graduate every year in the larger cities, a considerable majority settle in the city in which they train. Another difficulty in the smaller towns is that almost as soon as a nurse settles down to her work she gets married.

### BLACK SPOTS AT HUDDERSFIELD.

The President at the annual meeting of the Huddersfield and District Victoria Sick Poor Nurses' Association, in moving the adoption of the report, congratulated the members upon the good work which had been done by the nurses, and said that there were two black spots which it was best to face. The first was, as usual, finance, the expenditure for the year having exceeded the income by £54 18s. 7d. The other black spot was that they had had changes on their nursing staff. With regard to the former, the committee were able, owing to the savings put to a suspense account, to clear off the deficit and start the new year with a clean sheet. This is all very well, but, of course, it is not satisfactory. The expenses of the 12 months should be covered by the receipts of the 12 months, and with such a band of helpers as the Huddersfield organisation is able to command this should not be at all difficult. Changes in the nursing staff cannot be avoided, and may be due to various causes. So long as those employed are capable as well as fully trained the essential conditions are fulfilled, although it is naturally a matter for regret when a nurse who is much esteemed leaves a district. The question of the Queen's nurses at Huddersfield visiting school children is under the consideration of the committee.

### SHORT ITEMS.

It has been decided to asphalte the tennis court at the Auckland Hospital, New Zealand. A large amount of the sum required, namely £100, was raised by the nurses.

### The Nursing Outlook.

"From magnanimity, all fear above;
From nobler recompense, above applause,
Which owes to man's short outlook all its charm."

### ETHICS AND RESPONSIBILITY.

THE tendency of the present day is so much in the direction of seeking individual progress, or what is regarded as progress and advantage, that the struggle for existence tends to become less and less attractive, and more and more harmful to the formation of character, in the highest and best meaning of that word. The trend of matters is curiously obvious to the trained observer who notes the attitude of the "have nots" to the "haves." The possession of knowledge, money, or influence, or the reputation of such possessions, causes the "have nots" to pay homage to the "haves." The kind and amount of homage offered by the one to the other, in the course of the ordinary business of the world, usually constitutes a barometer for the observant, whereby they can tell where each stands, at the moment, in the general estimation. Indeed, the conduct of those who desire to possess or to have, constitutes them the stormy petrels of existence, and so, however much their conduct may be resented, such persons in fact may fulfil a useful purpose for the wise.

What then are the personal relations which should subsist between all workers, whatever their relative positions, in hospitals and kindred institutions? Clearly the nature of the work should sweeten the character and render those relations unusually pleasant and helpful. It is recorded of the late Dr. Temple that a Rugby boy said of him, in answer to certain criticism of the late Archbishop, that "Temple was a beast but he was a just beast." It would be well for every great establishment if the same statement could be truly made of its chief official. Each hospital community is in fact a little republic, and unless the principal officials are animated by a spirit of justice, and have enough character to insist, that they shall see only with their own eyes and hear only with their own ears, and so govern the institution, efficiency and happiness are not likely to prevail within its walls. The same principle applies to all who may be in authority. It is wonderful how valuable an asset a quiet, kindly manner, backed by a firm will, becomes in institutional work. The sister who knows her work, and the work of all under her charge, may secure the maximum of efficiency, whilst her wards work with the smoothness and regularity of a watch. Yet her individuality never thrusts itself into prominence. Such a sister's influence affects the charge nurses and makes the life of a probationer, despite the strain of the work, something to be always remembered with satisfaction. Good manners, a deliberate apprehension of the rights and feelings of other people, and an appreciation of the power of quiet firmness and kindly influence, may make life in an institution almost ideal, whilst they raise its reputation to the highest pitch. We take it in these days, where so great a point is made of the higher education of matrons and nurses, that one result should be to introduce these principles into all relations of hospital life.

As the facilities for travel increase the number of visitors to every hospital of importance increases too. The volume of correspondence relating to inquiries of all kinds is growing every year. It therefore becomes of some importance to consider what are the ethical relations which should prevail in such matters, especially where the officials concerned are, more frequently than not, oppressed by work of various kinds. We believe it would be an immense service to all concerned, if a general agreement could be come to, to require that each visitor coming from some distant place or country should bear a letter from the authorities of one of its chief institutions. It is clearly wrong that the time of overworked officials should be taken up by showing round all sorts of people who may come from mere motives of curiosity, who have little knowledge of or interest in the subject, and who therefore have no right to expect facilities to be given them at a busy institution to spend an hour or two of their time there when travelling. Of course, as between official and official, correspondence may prove most helpful. Indeed we should say, that however busy an official may be, there is no work so calculated to repay the worker, as that which keeps him in close touch with all who are actively engaged in similar undertakings. Knowledge begets knowledge, courtesy secures courtesy, and both help the worker to live rather than to exist. Therefore we believe strongly in constant inter-communication between all workers, in responsible positions, everywhere, throughout the world.

We should also like to see more reciprocity between the conductors of the more influential and reputable of technical newspapers. It would help the cause of sound administration and improved nursing to promote a spirit of generous recognition and co-operation between journalists in regard to these matters. After all a newspaper which is to have any weight must be measured as to its influence by the success with which it is conducted. Amongst the editors of reputable journals there is a tacit understanding that every contemporary should receive generous recognition at the hands of its fellows. This rule should certainly dominate the conduct of all Anglo-American nursing journals of repute. It is happily the exception to find a journal of nursing allowing a speaker to quote favourable comments upon itself from a contemporary, without indicating the source from which they emanate.

### Medical Electricity and Light Treatment.

By KATE NEALE, Sister-in-Charge of the Actino-Therapeutic Department, Guy's Hospital.

### III.—GALVANISM AND FARADISM

(*continued from p.* 269).

### 2. FARADISM.

THE account in the last number of THE HOSPITAL may have seemed a little lengthy, but you may be reassured for what you have learnt in connection with galvanism will serve you equally well for faradism, more especially as regards methods of treatment. These are so similar in the two cases that I shall mention here only points of difference. The instruments, however, are radically different, and some details on this point are necessary.

The essential feature of faradic treatment is this: whereas in galvanism you send a current that flows steadily and continuously, in faradism you employ one that is frequently and rapidly broken; that is to say, it flows for a fraction of a second, then stops, then reflows and again stops, and so on. A good faradic apparatus will make and break the current in this way from one hundred to two hundred times a second. Now it is quite clear that you could not possibly make and break a circuit so quickly by hand, and a special apparatus has been devised to effect this automatically.

*Apparatus.*

The principle on which a faradic apparatus works is rather complicated, and therefore a little difficult to understand. Let us begin with a simple experiment or two which will serve to make matters clearer. If you pass a current along an electric wire coiled round and round a thick rod of soft iron, the iron core will quickly become a magnet, and so remain until the current ceases to flow. Next wind a second and longer piece of very fine wire in a coil, but without any iron core in it at all. Bring this coil which we will call the Secondary Coil, near to the one with the iron core, which will be the Primary Coil, so that they lie parallel to each other, their ends touching. Now hold the two ends of the wire forming the secondary coil between your fingers and send a current along the primary wire; you will feel a distinct but short electric shock. Allow the current to continue to flow and you will feel nothing. Lastly, break the primary current, and again you will notice a momentary shock. If you repeat the experiment without the iron core inside the primary coil, you will still experience the same shock, but it will be of less intensity. In other words the passage of a constant current along a primary coil on make and again on break induces a momentary current in the secondary coil, and these induced currents are stronger when the primary coil encloses an iron core. Here then we have a means of producing a faradic current, for if we can devise some method whereby the current in the primary coil can be made and broken, say 100 times a second, we shall get every second, 100 induced currents in the secondary coil, and these induced currents will serve to apply to the patient as a faradic stimulus. A suitable method has been devised, and the whole apparatus is known as an "Induction Coil." Its current, though usually called faradic, after its discoverer Faraday, is sometimes spoken of as an "interrupted current."

*Induction Coil.*

Fig. 3 illustrates a faradic apparatus or induction coil. It consists of a box divided into three compartments, the middle one containing as a battery an electric cell (in this case a bichromate cell), to provide the current, while the right hand one encloses the induction coil, which, by means of a hinged end to the box can be lowered to a horizontal position as shown in the fig. On the left is a division for storing electrodes, wires, sponges, etc. The current from the cell is conveyed by two wires to the terminals *a* and *b* of the primary coil; *c* is the arrangement for rapidly making and breaking the circuit. It is known as a Vibrator because as soon as the current begins to pass it vibrates rapidly to and fro, giving rise to the well known buzzing of these instruments. The primary coil is inside *d* the secondary coil, which has its terminals at *f* and *g* (note between them

FIG. 3.—INDUCTION COIL.

the letter *s*, *i.e.*, secondary), *h* is the metal core, which can be drawn out or in according to the strength of current required.

To start the apparatus working, the rod shown in the figure projecting from the top of the cell is pushed down as far as it will go. This completes the circuit, and you should hear the vibrator begin to act at once. If it fails to, as sometimes happens, a brisk tap with the finger will suffice to set it going. Screw your wires to the secondary terminals *f* and *g*, and to their free ends attach whatever electrodes you are going to use. If you find the current too strong when you test it on yourself, withdraw the core *h* until the desired strength is obtained. Treatment is sometimes ordered to be given from the primary coil in place of the secondary, and the electrode wires must then be joined not to *f* and *g*, but to *e* and *f*—the letter *p* (primary) stamped between them denotes this.

When the treatment is ended unscrew the electrode wires and draw the rod out of the cell again as far as it will come. Neglect of this precaution will very soon exhaust the battery.

*How to Treat.*

Treatment by faradism is applied in much the same manner as by galvanism. The same forms of active electrodes are used, and an indifferent electrode is placed at the neck or elsewhere. The application may be either labile or stabile (usually the former), but no distinction is drawn between anodal and kathodal treatment, because a peculiarity of faradic currents is that both electrodes possess the same qualities, and it is therefore immaterial which is placed on the diseased part. If, however, you are making use of the primary coil for your current, and not the secondary, anodal or kathodal application may be ordered, in which case it is advisable to ascertain which wire is the anode, and which the kathode, by one of the tests given in previous chapter.

*Dangers of Galvanic and Faradic Currents.*

It is not often that any serious consequences follow the application of galvanism, provided a current of proper strength is used; but many patients, especially children, look forward to their treatment with considerable fear and you must do your best to reassure their timidity. Especially trying to these people is the first application of the electrodes and the sudden shock so produced. But this unpleasantness can be easily done away with if you make it a custom to bring both electrodes in contact with the patient before switching on any current and then slowly turning the handle of the collecting dial till the requisite strength is obtained. If this be done no shock should be felt at all. The same precaution must be taken at the end of a treatment, when you should switch off at the collecting dial before lifting the electrode from the skin. Be particularly careful over this when you are called on to apply galvanism to the head, because any sudden interruption of the current will be liable to produce serious giddiness.

Burns I have already alluded to as a result of bringing bare metal in contact with the skin, but such accidents can only occur as the result of carelessness. If any should appear I need hardly say that it is your duty to draw the doctor's attention to them and to ask what dressing should be applied, for they are often very intractable and take many weeks to heal.

*Diseases Commonly Treated by Galvanism and Faradism.*

Broadly speaking treatment by galvanism is usually selected for painful conditions (*e.g* neuralgia), while faradism is adopted when a stimulating effect is required (*e.g.* paralysis), but there are many exceptions to this rule. As regards galvanism alone it is commonly said that anodal treatment is more sedative than kathodal, but you will find that some physicians prefer kathodal treatment as a routine measure, while others believe the greatest benefit follows anodal application.

Still this is a matter into which it would be out of place to enter here as it does not greatly concern a nurse who will take her instructions from the doctor, but whatever variety may have been ordered you should always administer it yourself and never under any circumstances leave the treatment to the patient. Such slackness is to be deprecated on every ground. The patient knows nothing of the methods of treatment and as often as not wearies of the work and loses faith if improvement does not follow as rapidly as he had hoped. These failures would be much less likely to occur if you conscientiously applied the treatment yourself, and you can do much to insure success by encouraging the patient to look forward to a successful issue to his case. Remember that it often takes at least a month for any appreciable progress to be made, even if treatment be given regularly, and with customary frequency—three times a week.

It would serve no useful purpose to give lists of the many diseases in which galvanic or faradic electricity has been used, but it will be of some assistance to you to know what are the principal complaints you may be called on to treat.

Of all diseases those of the nervous system respond most satisfactorily to electricity. Hysteria in its many guises (paralysis, spasm of muscles, aphonia) will probably form a large percentage of your cases, while examples of hemiplegia, writer's cramp, locomotor ataxia and neurasthenia will come under care from time to time. Lead poisoning and other forms of neuritis—*e.g.* arsenical, gouty, and alcoholic—often need long and careful treatment before any amelioration is observed. Facial paralysis is another common condition you will see, and special care is required in its treatment because of the danger noted above of applying currents to the head. Children are often afflicted with paralysis (infantile paralysis) of one or more limbs, usually the leg, and they will require treatment extending over months or years.

*(To be continued.)*

### The Nurses' Clinic.

### FEEDING PATIENTS. BY A SISTER.

"You may take a horse to the water but you cannot make him drink," is a proverb the truth of which nurses realise every day. "You may bring the food to the patient but you cannot make him take it," is the nurse's version. And unfortunately the difficulty is always greatest where the patient's chance depends on his ability to take nourishment. What to feed the patient with is generally the doctor's business; how to get him to take what the doctor has ordered is the nurses'. Much has been written about daintily-served meals and variety of food, but I am dealing rather with hospital patients, where the nurse has no choice but to give the food ordered, and to use the hospital crockery, and with acute cases of illness where two-hourly feeds of milk represent the "dainty meal." Very often refusal to take food is due to the condition of the patient's mouth—hot and dry, with a glazed cracked tongue. All secretions are lessened where there is a high temperature; the mouth is no longer moistened and more or less cleansed by the constant flow and swallowing of the saliva, the spout of the feeder sticks to the parched lips, and suction is performed with great difficulty. But if the patient is allowed to rinse his mouth before each feed with water into which a few drops of lemon have been squeezed, or, if too helpless to do this, his mouth and lips are moistened with a bit of wool or lint dipped in the lemon-juice and water, drinking will be a far easier matter. It is even more important to repeat the process after each feed. It certainly takes time, and time is a very important

THE NURSES' CLINIC—*Continued.*

consideration in a busy ward, but no one who has not experienced it can realise the misery of a cracked dirty tongue, and the offensiveness of decomposing milk in a dry mouth, hardly able to swallow, and stiff with the lack of saliva. Like Dives, the poor patient lifts up his eyes, being in torment, and longs for Lazarus to dip his finger in water to cool his burning tongue. One great mistake nurses sometimes make is to give the stimulant ordered with the food; very often the patient has a great dislike for brandy, and if his milk contains it he cannot be persuaded to drink it. If there is a great distaste for brandy the doctor can prescribe other forms of stimulant, but it is not such an easy matter to find a substitute for the food which the nurses' thoughtlessness has led him to refuse.

"Two-hourly feeds" should be given two-hourly, and on no account should the feeding-cup be left on the locker to be sipped at occasionally. It is a good plan, however, when the nurse has several two-hourly feeds to give, to let one patient take as much as he will at a time, and then to leave him for the next, returning to him a few minutes later, when he will be more likely to finish his feed.

Sometimes a patient has a dislike to a feeding-cup, but will take his milk from a medicine glass — especially if the glass contains only the amount he will drink at a time. When it is a case of feeding by drachms, instead of ounces, a minim measure is often easier to manage than a teaspoon, and there is less likelihood of the food being spilled, a glass two-ounce medicine measure with a spout like a feeding-cup is better still, though it is a luxury not found in every ward. When a patient is unable to feed himself, the nurse should pass one hand and arm under his pillow, raising it and his head together while she holds the feeding-cup in the other hand. One thing nurses sometimes forget is that a patient often wants some plain water to drink in addition to his two-hourly feeds of milk, milk and soda, or milk and water as the case may be. Where he is allowed cold drinks his feeds should be measured into a cup and stood in the refrigerator on the ice, rather than being taken as required out of the general ward supply. If there are several patients on two-hourly feeds a larger cup or mug will be needed that is all, which should be prepared as the previous feeds are finished. Some doctors never allow ice to be sucked nor put into food, for fear that the ice may be taken from impure water. Also a small piece does not cool the drink and a large one dilutes it too much. If cold water cannot be got by letting the tap run till the water which is in the pipes has run off, some should be drawn and put into the refrigerator, where a syphon of soda water also should always stand. Sometimes a patient either cannot, or will not, open his mouth and the teeth remain tightly clenched, a condition described as trismus. Nasal feeding is then usually ordered, but it is very often possible to feed a case of this kind by the mouth, for nearly always one tooth at least is missing and a small rubber tube can be passed through the gap, the other end having been previously stretched over the spout of the feeder. If a few drops of olive oil or a scrap of vaseline are put into this end of the tube it will slip very easily over the spout, this is also a help when putting tubing on a gag. Great care must be taken, when a patient is fed in this way, not to pour too quickly at first, in case the patient should be unable to swallow, not to pour the feed in a continuous stream, and to be sure that his head is not too low, or a choking attack may result. If a patient is in a semi-conscious condition he will often hold fluid in his mouth for a considerable time before swallowing it if he makes any attempt to swallow at all. Holding his nose for a few seconds between the finger and thumb will often produce the desired result, or tickling the throat just above the thyroid cartilage with the finger and he will swallow what he has in his mouth automatically. Babies often fail to take their bottles because they are given a teat with too small an orifice, a weakly baby has not much power of suction and requires a different teat to a strong baby if he is to get the milk out of the bottle. And, naturally, if a strong baby has a teat with too big a hole he will get too much milk at a time and take his feed too quickly with a great deal of choking in the process.

### Incidents in a Midwife's Life.

### IN THE LIVERPOOL SLUMS.

Two a.m. "Oh, dear, whatever is it now? That door again!"

Nurse C—— scrambles out of bed, slips on her slippers, struggles into her dressing-gown whilst running downstairs, to find thrust upon her that inevitable red card, the property of the Ladies' Charity, and the summons to duty.

Five minutes later she is off, bag in hand. That sizeable bag with its latest additions, registered C.M.B., the pride of every young midwife, because it makes her feel so important; the bane of Mrs. Gamp, because she does not see the need of it; and the butt of the street arab, who smirkingly inquires, "Got twins in there, Nurse?" Hurriedly she rushes through the dark streets to three Court, five house, B—— Street, to inquire for the "Cellar lady"; down the steps she scrambles nearly breaking her neck, to find half the ladies of the court assembled to assist her. In fact, they have been doing a little assisting on their own, as the patient's flushed appearance tells.

"Now, off with you, I only want one," says nurse briskly, upon which arises a heated discussion, as to who should have the honour of being lady-in-waiting, each one claiming kinship to the patient and asserting her right, till finally nurse bundles them all out. Mary Ellen sneaking in a few minutes later is retained.

"Hot water!" says nurse. Of course there is no hot water, and, what is more, there is no kettle; an empty corn-beef tin, which looks as if it had done duty for years as a kettle and teapot, the presence of tea leaves bearing witness to one of its functions comes to view, and is placed on the fire.

Nurse turns her attention to the bed, feeling carefully round among the old skirts, coats, and other litter. Then she comes on sundry arms and legs, and drags to view various dirty, scantily-clothed children. Mary Ellen carries them off to obliging neighbours, with injunctions from nurse to bring back with her various articles which are needed but not to be found.

Whatever else they are, the poor are invariably kind and open-handed to one another at such a time, so that nurse has no misgivings about being able to borrow. She begins to set the bed in order, but there is no mackintosh—no such luxury. A pile of newspapers supplies the deficiency, and baby when he arrives and has rested for a few minutes on this luxurious couch, turns out a walking advertisement, with "Houses to Let," the names of quack medicines, and what not stamped legibly all over his dear little back.

Mary Ellen returns with the information that Sarah Ann has lent the tin-dish that contained the hot-pot at Christmas. So a little later on that very same dish becomes baby's bath, and contains a decidedly livelier hot-pot.

Business is in full swing. Nurse has wrapped baby up and put him at the bottom of the bed, out of the way while she attends to mamma. A little later she misses him, and turns round to find Mary Ellen and grandma, who has crept in, cramming butter and sugar down the poor mite's throat. "Sure, Nurse, Mrs. Gamp allus gave it to my children, and she has nursed for years and years and years," says grandma, in reply to nurse's indignant remarks. "Well," nurse reflects, "it might help to keep him warm, if it were rubbed outside the poor mite," for the old pillow-case which is the only available article to wrap him in will not do much in that direction. The mother, when questioned, says she had to put the baby clothes in pawn, to get food, a week ago, and has not been able to get them out since.

Everything is finished, the mother as comfortable as possible, and baby contentedly sucking his fist, so nurse prepares to go home. "But what about the relief ticket, nurse?" comes the eager query. The charity to which nurse belongs allows a ticket granting the recipient a certain amount of groceries, bread and coal, and this ticket is anxiously inquired after the minute the patient gets time to speak. Nurse promises to bring the ticket when she comes during the day, but that does not suit the mother, she wants it now, this very minute, and launches forth in a long tale of woe, as how she has not had a bite to eat, only a sup of tea, and she had to pawn her boots to get that. Nurse has heard that tale repeatedly and knows its worth, but this case seems deserving, and nurse is anxious that her patient should have some nourishment, so Mary Ellen is sent off to get a pint of milk, and spend sixpence in sundries. In a short time she returns with the milk, the sixpence she has spent in a four-pound loaf with a penny bun to make weight, half-a-pound of margarine, two ounces of tea, and had three farthings change.

Nurse warms the milk, gives some to her patient, and with final instructions that none of the ladies of the court are to be allowed in, takes her leave.

### Surgical Nursing in Switzerland.

BY A CORRESPONDENT.

For several years I have nursed for a well-known surgeon in Switzerland. In my humble opinion the surgery here is beyond ours, and the nursing far behind. Things on the whole are simpler, but the results are more satisfactory. The surgeon I mention is a specialist for women, and I should like to give one or two details with regard to the preparation and the treatment for the first 24 hours after an abdominal section.

The operation takes place at 9.30 A.M., the day before an aperient is given, in the morning, usually liquorice powder, enema later on if necessary. The food during the day is only liquid and the last nourishment is taken at 7 P.M., some good "bouillon" with an egg beaten up in it, in the morning nothing whatever, no stimulant. The patient takes an ordinary warm bath, no soda or extra scrubbing. At 9.30 A.M. the surgeon comes in and, after speaking encouragingly to the patient, I have often seen him take her in his arms and carry her downstairs himself to the *chambre à endoemir!* The anæsthetic given is always ether, unless for any special reason chloroform is an absolute necessity; a mixture is never given, neither have I ever seen gas used. As soon as the patient is unconscious the surgeon and his assistant begin the disinfecting. The only thing occasionally done by the nurse beforehand is the shaving. The abdomen is scrubbed with soap and water, then with alcohol and ether, and lastly with "sublimé." The details of the operation itself I do not feel competent to give, I will only say that it is performed with lightning rapidity and with the very strictest antiseptic precautions. The dressing consists of iodoform or vioform gauze loosely placed over the wound, and then a strip of the same brushed over with collodion—an exceedingly good dressing as one has no fear of it slipping. Plenty of wool and an ordinary flannel binder are then applied. The binder is a perfectly straight piece of double flannel, kept in position by strips of the same passing under the thighs and pinned in front. Now the patient is ready for bed, the night-dress having been changed, a piece of wool put over the chest, and any damp parts of the body dried. In bed a pillow is allowed, and a bolster always placed under the knees; cradles are not used. Towards 5 or 6 P.M., if the pain from flatulence be very severe, an enema of camomile is very gently given, and it is often a great relief. Later, towards 10 P.M., when, if the case is going as it should be, the surgeon pays his last visit, he usually turns the patient on to her side, arranging a pillow at her back, and, if he thinks it necessary, gives an injection of morphia. About 2 A.M. the first drink is allowed, and consists of sips of peppermint tea—boiling water poured over dried peppermint leaves—splendid for flatulence, and, according to my patients, very refreshing. Enemas of camomile are given from time to time, until the flatus passes freely, and only very light liquid food; then some soups, beef-tea, with egg, nothing solid until after the first motion has been passed. If the patient is very weak after the operation no stimulant is given by mouth, only enemas of Bordeaux, or caffein subcutaneously, or subcutaneous transfusion of salt and water. The stitches are always removed on the tenth day, and after removal the wound is again covered with gauze and collodion. About the third day the patient is lifted from the bed so that it can be thoroughly made, and in about a fortnight is allowed to get up. Imagine a double ovariotomy up and walking in so short a time. They are also allowed to move and turn in bed a great deal sooner than with us. The patient is always taught, before operation, to pass water lying down, thus, in most cases, avoiding the use of the catheter. For giving ordinary enemas a Higginson's syringe is never used, always a douche can.

### Resignations.

Foundling Hospital.—Miss Ethel Tawney has resigned the post of infirmary superintendent at the Foundling Hospital, having been appointed health visitor to the Municipal Borough of Croydon. Miss Tawney was trained at the Hospital for Sick Children, Great Ormond Street, and has held her present post for over six years. She holds the certificate of the Sanitary Inspectors' Examination Board and that of the National Health Society.

### To Nurses.

We invite contributions from any of our readers, and shall be glad to pay for "Notes on News from the Nursing World," or for articles describing nursing experiences at home or abroad dealing with any nursing question from an original point of view, according to length. The minimum payment is 5s. Contributions on topical subjects are specially welcome. Notices of appointments, letters, entertainments, presentations, and deaths are not paid for, but we are always glad to receive them. All rejected manuscripts are returned in due course, and all payments for manuscripts used are made as early as possible after the beginning of each quarter.

### Notes from India.

BY OUR OWN CORRESPONDENT.

Nurses are badly needed in India, and by nurses I mean really good English-trained women with knowledge and intelligence. Here the nursing work, with a few exceptions in civil and general hospitals, is done almost entirely by Eurasians, who may, up to a certain point, be fairly good nurses, but are not exactly capable women. They are country born and bred, have much of the instincts of the darker race in them, and, though speaking English and dressing as Europeans, fail to impress one as possessing the reliability necessary for their work as nurses. They are lazily inclined, and have little sense of strict truthfulness and honour.

Civil and general hospitals in large cantonments and cities employ these women as nurses, with probably a Eurasian matron at their head. They are less expensive in the matter of pay, as they make the rupee go much farther than any English woman could do; they have a complete knowledge of the language of the country, the proper price of food, etc., and are valuable in many ways in managing the native patients.

In all this they have the advantage over the English nurse, but in nothing else; for wherever an English-trained nurse has taken over the duties of a matron, following the reign of a country born and bred woman, there has been progress and a happier state of affairs generally. There are hospitals in nearly all the hill stations in India; but, as there are very few people comparatively in the cold weather months, many of them are closed from about November to March.

In small hill stations a matron may be kept, who for a small salary of 40 to 50 rupees a month will look after any paying patients and manage the hospital generally, and, with the assistance of only an ayah, do good work. In larger places, where there is a staff of Eurasian nurses, things are run on a larger scale and with more expense.

The Up-Country Nursing Association sends nurses out to all parts of India, chiefly to midwifery cases, though there are frequently cases of enteric among officers' wives and children who call in their services. The Association has a staff of about nine nurses, but they are badly in need of money. For a long time only Eurasians were employed, but they proved so far from satisfactory that the committee of management tried slowly and surely to improve matters. Now there are only English-trained nurses. Their pay is about 30 rupees monthly, and they are maintained in the home. When out at cases their fee is 5 rupees a day, which goes to the upkeep and expenses of the Association. It will be seen how small the number of nurses this establishment is capable of employing owing to the lack of funds, and one can readily imagine that in this large area, where sickness and suffering are inevitable, and so many tiny lives are brought into the world, that skilled and careful work is required and also an increased number of willing hands. Money is being solicited in some of the overland newspapers for the Association and it is to be hoped that there are many in India and at home who will support this good cause.

### Death in Our Ranks.

Eastbourne Borough Sanatorium.—We regret to announce the death on Friday last, from heart-disease, of Miss Elizabeth Walker, sister at the Borough Sanatorium, Eastbourne. Miss Walker, who was 38 years of age, had been ill for six months. She was greatly esteemed in the institution

### Central Midwives Board.

CHARGES AGAINST MIDWIVES.

A special meeting of the Central Midwives Board was held on Tuesday afternoon, July 25th, to consider charges alleged against certain certified midwives, against whom *prima facie* cases had been made out. There were present, Dr. Champneys (in the chair), Dr. Dakin, Mrs. Latter, Miss Paget, Mr. Parker Young, and Miss Wilson.

### The Case of Annie Blightman.

Annie Blightman was first called. The charges against her were—

That she had been guilty of negligence and misconduct in attending confinements and acting as a midwife without taking with her the appliances and antiseptics required, that she did not keep a register of cases, and that she did not keep herself scrupulously clean in every way.

Dr. Watts, Medical Officer for Farnborough, was the witness against her, and he stated that he had read the rules to her and told her she must get antiseptics and appliances, allowing her however to attend the next case without the appliances if she were unable to get them. This case did not occur until 12 days after Dr. Watts had seen Mrs. Blightman, but she had not got the antiseptics. Dr. Watts said that Mrs. Blightman's hands and nails were extremely dirty, and that part of the afterbirth was left behind, and it appeared that Mrs. Blightman did not know any real test for ascertaining whether all the afterbirth had been removed. The patient had puerperal fever and died. Dr. Watts was of opinion that Mrs. Blightman should be removed from the lists of certified midwives as being unfit to practise as such, especially as he pointed out that her livelihood was not dependent on it.

In the course of the evidence, it also appeared that she did not know how to take a temperature. Mrs. Blightman was herself anxious to have her certificate cancelled, as she felt herself quite unable to conform to the rules of the Board. She admitted that her hands were particularly dirty that day, but said that she generally cleaned them well, and produced testimonials testifying to her cleanliness. With regard to the purchase of the antiseptics, she stated that as the confinement was expected daily, she was unable to go for them, as the nearest drug stores were four miles distant. Dr. Watts interposed that she could have got them at the chemist's 1¾ mile away only. She had acted as a midwife for 24 years, and this was the first death which had occurred. With regard to her livelihood, her husband was an old man, and would soon be pensioned at 7s. a week by the railway company for whom he worked. The Board, after consideration, felt obliged, though sympathising with her case, to cancel her certificate, and held that the first two charges against her were proved. The chairman said that in the matter of cleanliness, it was necessary to point out the difference between ordinary personal cleanliness and surgical cleanliness, and that it was in the latter aspect that Mrs. Blightman might be considered to have been at fault. He recommended her in any future work to use antiseptics plentifully.

### The Case of Mrs. Batchell.

Mrs. Batchell was then called. The charges against her were as follows:—

That being in attendance at a confinement on June 15th and subsequent days, she was guilty of negligence and misconduct in the following respects: When called to the confinement she did not take with her the appliances required. She did not disinfect her hands and forearms before touching the patient's genital organs. Before making the first examination she did not wash the patient's external parts with soap and water, and then swab them with an antiseptic solution. On the occurrence of a shivering fit in the patient she did not advise that a registered medical practitioner be sent for. She did not notify the local supervising authority of the necessity of sending for medical help.

Mrs. Eldridge said the baby was born on Thursday evening at 6.35, and that Mrs. Batchell did not come till 6 p.m. on Friday; at 2.40 on Saturday morning Mrs. Tully had a shivering fit. Mrs. Batchell came on Saturday afternoon at 2 o'clock and said she thought the fit was due to the milk coming. Mrs. Tully was, however, much worse on Sunday, and Mrs. Eldridge sent for a doctor about 12 o'clock, but could not get one. Later in the afternoon Mrs. Batchell came and suggested that Dr. Easton be sent for, and he came about 5 o'clock. Mrs. Eldridge accused Mrs. Batchell of not having kept her sister-in-law thoroughly clean, and of having no antiseptics or appliances.

Dr. Easton then gave evidence, and said he found the patient's temperature, when he arrived on the Sunday afternoon, to be 105 and her pulse 160. Part of the afterbirth had not been removed, and was taken away on Monday by Dr. Easton. Subsequently an abscess formed on one of the buttocks, and the patient was sent to the infirmary, and was now doing well. For the defence it was stated that the absence of antiseptics and appliances was due to the fact that Mrs. Batchell had had no time to bring them as she had been suddenly called to another case that day. With regard to the shivering fit it was not impossible that it might be caused by the milk coming, though it was rather soon. She was unable to read or write, but her daughter had read the rules to her. She had been 26 years acting as a midwife, and had successfully attended most difficult cases.

The Chairman said that they had gone most carefully into the question, and it was with the deepest sympathy for her case that they felt obliged to cancel the certificate. They, however, only had the administration of the Act in their hands, and they had to safeguard the mothers of England. They thanked the solicitor especially for elucidating the case, and he replied that though he felt deeply for his client, he could not see that the board could come to any other decision.

### The Case of Miss Mugbridge.

The charges against Miss Mugbridge were—

That on April 25th, 1905, being in charge of the Birkenhead Maternity Hospital as temporary matron, during the absence of the matron, she was intoxicated, and, by reason thereof, her conduct and demeanour caused alarm to the patients in one of the wards. That on April 26th, 1905, she went out of the hospital at 5.30 P.M., leaving a patient at that time in labour in the charge of nurses who were all probationers and uncertified, and that, on returning to the hospital at 7 P.M., after the patient had been delivered, she was found to be intoxicated.

Miss Spenser, a nurse at the hospital, was the witness against her, and said that, on hearing the charges brought against her at the hospital by herself and another nurse, the Hospital Committee had summarily dismissed Miss Mugbridge. Miss Spenser recapitulated with details the charges against her, and Miss Mugbridge had written a letter to the Board pleading for mercy and not denying the allegations. Her certificate was cancelled.

### The Case of Elizabeth Brown.

The case of Elizabeth Brown was then considered. The charges against here were—

That she was in a state of intoxication while in the performance of her duties as a midwife. That, being in attendance, she did not disinfect her hands or forearms before touching the genital organs of the patient. That she did not, before making the first internal examination, wash the patient's external parts with soap and water, and then swab them with an antiseptic solution. That she did not, on rupture of the perinæum of the patient, advise that a registered medical practitioner be sent for. That in another case she was in a state of intoxication while in the performance of her duties as a midwife. That being in attendance she did not disinfect her hands or forearms before touching the genital organs of the patient. That she did not, before making the first internal examination, wash the patient's external parts with soap and water, and then swab them with an antiseptic solution. That when called to a confinement she did not take with her the appliances required by the Act. That she did not keep herself scrupulously clean in every way as required by the Act.

The evidence against her was based on statements by the medical officer and the Midwives' Inspectors. The two patients concerned had made signed statements against her, but subsequently wrote revoking these statements. Her certificate was cancelled.

### The Case of Anne Homer.

After hearing the following charges alleged against Sara Anne Homer, *i.e.*:

That, being in attendance at a confinement on May 16, 1905, she was then guilty of negligence in the discharge of her duty as a midwife. That, while in attendance, the patient being seriously ill, she did not advise that a registered medical practitioner should be sent for. That, the mother having died before the arrival of a registered medical practitioner, she did not notify the death to the local supervising authority.

The Board decided to severely censure Sara Anne Homer. Dr. Jackson, the medical practitioner who came after the death of the patient, wrote a letter saying he thought that the midwife had in other respects done her duty.

### The Case of Mary Slater.

Mary Slater was accused as follows:—

That being in attendance at confinement on June 1st, 1905, and subsequent days, she was guilty of negligence and misconduct. It was alleged that before making the first examination she did not wash the patient's external parts with soap and water and then swab them with an antiseptic solution. That she did not wash the patient nor change her clothes until June 3rd. That on the rupture of the perinæum of the patient she did not advise that a registered medical practitioner be sent for.

As there was ample proof of these charges and Mary Slater had been severely reprimanded in November last on similar charges her certificate was cancelled.

### The Medico-Psychological Association.

### THE PRESIDENT ON NURSING ORGANISATION

The sixty-fourth annual meeting of the Medico-Psychological Association was held last week. The new President, Dr. T. Outterson Wood, devoted a large part of his address to the question of nursing. There were, he said, now 6,900 nurses possessing the certificate of the Medico-Psychological Association, probably the largest number in the nursing world holding certificates of uniform value from one constituted authority. Last year the period of training for that certificate was fixed at three years, thus freeing the Association's nurses from the taunt of being inadequately trained as compared with hospital-trained nurses, and it would greatly strengthen their claim for inclusion in any scheme of State registration. He deprecated applying the term "attendants" to male nurses in asylums, to whom the term "nurse" was as applicable as to females. Some advocated female nurses for male insane patients, and he thought that some such cases as were physically ill might be suitably nursed by women, but the proportion could not be large. It must not be forgotten that competent male nurses were as much an absolute necessity in asylum life as in private practice, and he could not help feeling that they were often what doctors themselves made them. Among hospitals generally there was great want of uniformity in the training given to the nurse, yet hospitals would not recognise the Medico-Psychological Association training—though in many respects it was better than their own—but compelled them to go through the whole course like a beginner. He considered that it would be well to follow the example of the Royal British Nurses' Association and organise a Registration Board as soon as possible. There were now only three organised qualifying bodies for nurses in the field with regard to State registration, namely the Royal British Nurses' Association, the Central Midwives Board, and the Medico-Psychological Association, and all that could be urged against the nurses of the latter being included in the proposed legislation was that they had not passed a Registration Board. He was not quite sure that all the stir now being made about State registration would be of much material benefit to the nurses themselves, or whether perking them up with a glistening pride might not end in their wearing a crown of sorrow, but there was no reason why the medico-psychological nurses should be made to appear outside the pale of official recognition. The public were well protected in the matter of nurses by the members of the medical profession, who took good care to employ such nurses as were properly trained, of good character, and well recommended. He doubted if any scheme could improve upon the excellent system of the Royal British Nurses' Association, under the presidency of Princess Christian, and he added that the Association was largely indebted to her Royal Highness for the interest she had taken in the status of asylum-trained nurses.

### Everybody's Opinion.

[Correspondence on all subjects is invited, but we cannot in any way be responsible for the opinions expressed by our correspondents. No communication can be entertained if the name and address of the correspondent are not given as a guarantee of good faith, but not necessarily for publication. All correspondents should write on one side of the paper only.]

### NIGHT NURSES AND LITTLE COMFORTS.

"E. W." writes: I quite agree with "M. E. F." that some comfort should be provided for nurses on night duty, and there are certain little attentions which, if shown, would greatly add to the enlightenment of the dreary night watch and also endear us to one another. We are called upon to show kindness and close attentiveness to those who are sick, and why should we not do so to our fellow-workers, and, indeed, to all with whom we come in contact?

### A QUESTION OF UNIFORM IN IRELAND.

Mr. H. B. Harris, J.P., of Spanish Point, County Clare, Ireland, writes: Referring to your paragraph in issue of July 1, under the heading "Libel Action by a Hospital Matron," allow me to ask who reformed the nursing system since Charles Dickens, in his book "Martin Chuzzlewit," immortalised Betsy Prig and Sarah Gamp? Surely it was not nurses of this stamp, nor men or women representing the nursing system of those days. All the reform came from laymen and laywomen representative of the type of the noble-minded lady who, during the Crimean war, made sacrifices in the interests of humanity. I submit most respectfully that unless committees of management of hospitals and infirmaries are sustained in their efforts to promote efficiency in every department of these establishments abuses will creep in, and perhaps another reign of Sarah Gamps and Betsy Prigs will again distinguish the future generation as on the occasion when Charles Dickens wrote. And allow me further to ask why should not nurses be as proud of their uniform as the officers of His Majesty's service are, and consider it an honour to appear at balls and other public functions like these soldiers, wearing their uniforms only? To me, a man in his 76th year, it would be more interesting to note a nurse in her uniform on such public occasions, thus showing who or what she is—more especially if it be a function connected with the institution for which she worked—than to see her clad in garments which cover the person so insufficiently as the so-called *décolletée* dress.

### THE UNTRAINED NURSE AS MIDWIFERY PUPIL.

"L.R.C.P." writes: Having a large practice in maternity cases, I have been in the habit of taking trained nurses as pupils and preparing them for the L.O.S. examination in return for their services. This arrangement has been an advantage to me, and a great help to those nurses who cannot afford to pay a large fee for instruction. Hitherto I have always had trained, educated, and disciplined women and the scheme has worked smoothly and well, and has resulted in a mutual advantage to both until the arrival of my last pupil. She was recommended by a previous pupil. I consented in order to oblige a former pupil, and arranged for her to come. She came to me in July 1904 and left this month. During the year she went up three times for the L.O.S., obtaining the certificate in February 1905. When she failed the first time I advised her to try again, and knowing her circumstances, with the best possible intentions I allowed her a small salary of 10s. per week. Again she went up and failed, and again I prepared her for the next examination. This time she was successful. I now explained to her that, having passed the examination, she would be able to obtain a post, but that I was in no hurry for her to leave. But, feeling she was now fully qualified and had her certificate, her conduct underwent a remarkable change: she commenced to quarrel with the household, gave herself airs, became rude and insolent. She actually drew up an agreement on her own account, in which she agreed to attend to the cleanliness of the instruments, keep the books, etc., on condition that she was taught "all that is required of a nurse, both practicable and theoretic." I refused to sign such an agreement, and explained that if she required an agreement I would give her one to copy and sign, which she did. Soon after this event I was laid up, and during my illness her conduct became unbearable. She quarrelled with my assistant, maintaining that she was not subordinate to my assistant, who was an M.D. and B.Sc., and gave in her notice to leave. This I accepted. Since leaving she has taken rooms near, and threatens to set up in opposition, and continues to annoy me in every possible way. After this experience I have come to the conclusion that every midwife should be a trained, educated, and disciplined nurse, which precaution would go far towards preventing a recurrence of such troubles.

### Appointments.

[No charge is made for announcements under this head, and we are always glad to receive and publish appointments. The information, to insure accuracy, should be sent from the nurses themselves, and we cannot undertake to correct official announcements which may happen to be inaccurate. It is essential that in all cases the school of training should be given.]

Birmingham and Midland Free Hospital for Sick Children.—Miss Annie T. Bell has been appointed matron. She was trained at the Evelina Hospital for Sick Children, London, and at Guy's Hospital. She has since been sister at the Evelina Hospital, and housekeeping sister at Guy's Hospital.

Blackpool Infectious Diseases Hospital.—Miss Edith M. Procter has been appointed matron. She was trained at the Nightingale School, St. Thomas's Hospital, and Cardiff

Infectious Diseases Hospital. She has since been matron of the Isolation Hospital, Llantwit Fardre, Pontypridd, matron of the Fever Hospital, Rhondda, and deputy-matron of Cardiff Infectious Diseases Hospital.

EAST LONDON HOSPITAL FOR CHILDREN.—Miss Maud Mellor has been appointed staff nurse for Light department. She was trained at the Victoria Hospital, Blackpool.

NEWCASTLE-UPON-TYNE UNION INFIRMARY.—Miss Ethel Breakenridge has been appointed charge nurse. She was trained at Fir Vale Infirmary, Sheffield. She has since done private nursing for the Midland Counties Institution, Birmingham, and has been sister at Aston Infirmary. She is registered under the Central Midwives Board.

THE GRACE SWAN MEMORIAL COTTAGE HOSPITAL, SPILSBY, LINCOLNSHIRE.—Miss Florence M. Bird has been appointed matron. She was trained at Stockton and Thornaby Hospital, Stockton-on-Tees, and the Western Fever Hospital, Fulham. She has since done private nursing at Stoke-upon-Trent, Cardiff, and London.

### Novelties for Nurses.

(BY OUR SHOPPING CORRESPONDENT.)

### FIRST-AID POST CARDS.

MR. F. REYNOLDS, of the firm of Reynolds and Branson, Leeds, ambulance experts, sends to the office a series of clever and amusing post cards, each one of which conveys a special warning what *not* to do in cases of emergency. Mr. Reynolds has lent his artistic skill to afford amusement, and at the same time he arrests attention and probably imprints a lesson on the memory which may one day be fruitful of good. The district nurse is glad of an acceptable means of conveying useful precepts. Mr. Reynolds would doubtless send her some first-aid post cards if she asked him for them.

### THE BABY CARRIER.

THIS is a simple little contrivance which we feel sure district nurses especially will like to hear of. Briefly, it is a little hammock or sling formed of string netting and attached to a strap of webbing provided with metal eyelet-holes. The nurse or mother throws the strap over her shoulder, places the baby in the sling and fastens the hook attached to the end of the sling in one of the eyelet holes. The baby lies safe and reposeful, and can be carried anywhere, leaving to the mother free use of hands and arms. It seems strange that so simple and practical a contrivance has not been in universal use long ago. I hear that it is becoming increasingly popular and as the cost is only 2s. 6d. no poor mother need be without it. It is sold by Messrs. S. Matthews and Co., 129 Duddeston Mill Road, Birmingham.

### NEEDLEWORK AND CHARITY.

NURSES are busy people and many of them must depend upon others for the making of their garments. There are, therefore, possibly amongst our readers some who may like to know where beautiful needlework is undertaken, the proceeds of which aid a deserving charity. The St. John's Hospital at Oxford is a home for the incurably ill. It is managed by the Sisters of St. John the Divine. Here both patients and sisters undertake the making of all sorts of underclothing, for grown up people and for children. They will make up materials sent to them or provide what is desired themselves. The cost is very moderate, and is regulated by the amount of work entailed. The good sisters also supply home-made marmalade and sweets. They are glad to send a price list to inquirers.

### CELLULAR CLOTHING FOR SUMMER WEAR.

I VISITED the Aertex Cellular Clothing Company at their depot at 417 Oxford Street recently, and was much impressed with the variety and excellence of the garments made of the material which is their speciality. This material may be described to those who are unacquainted with it as a fabric of woven holes. This arrangement permits of a circulation of air, which removes the objection raised against closely-woven cotton materials. The cellular fabric is porous, non-conductive of heat, light and durable. It is admirably adapted for the use of delicate people prone to catch cold easily. The finer kinds have a particularly attractive appearance, and these are, of course, the most suitable for summer wear. Every form of under-garment for men, women, and children is made by the company. The fashioning is simple or ornate, according to the taste of the purchaser. The workmanship is very good. We know from experience that those who adopt cellular clothing are its constant patrons and advocates. Cellular clothing is most comfortable, and forms an admirable substitute for woollen garments.

### Where to Go.

THE GREAT CENTRAL RAILWAY AND THE HOLIDAYS.—Every year the railway companies offer greater facilities to travellers in the holiday seasons. The Great Central Railway has some most attractive programmes which provide tours at wonderfully cheap rates. These programmes can be had on application to the Marylebone Station, or at any of the Company's offices. The trip to Cleethorpes-on-Sea costs only 4s. 3d. return, which works out at nine miles for one penny.

### TRAVEL NOTES AND QUERIES.

BY OUR TRAVEL CORRESPONDENT.

CYCLING IN BELGIUM (Nemo).—August is an expensive month, but three weeks can be managed well on the money at your disposal. Go to Ostend by the G.S.N.C. As you already know Bruges and Ghent, go on to Brussels, stay three days—one to see Brussels itself, one to visit Mechlin, and one to go to Louvain. Then on to Namur by an early train and take the steamer down to Dinant. If the river is very low the steamers do not run and you must take train instead. At Dinant (Hôtel Tête d'Or) you can stay ten days and will be able to see much of the beautiful country round. Do not omit a visit to Veve Celles.

A WEEK IN BELGIUM (A. W.).—It can be done on the money, but you must be content to remain in one place. Cross to Ostend by the G.S.N.C. and go straight to Bruges to the Hôtel Panier d'Or in the market place. Ask for a room on the third floor. Take early breakfast and late dinner in the hotel, and rely upon a confectioner wherever you may be in the middle of the day for luncheon. Courtrai and Oudenarde can be visited in one day very cheaply from Bruges. You must see the hospital of S. Jean in Bruges where Memling's most celebrated pictures are, also the Cathedral and the Church of Notre-Dame, and make a short excursion to Damme to see its old Hôtel de Ville.

BOATING, ETC., IN NORTH BRITTANY (X. Y. Z.).—I have nothing to add to what I said in my letter. It is risky for inexperienced people. The hotel your friend told you of in St. Malo is very good, but too expensive. The Poulardainé one is excellent and reasonable, but Mont. St. Michel is not a place to remain a week in, charming as it is; the young people would be bored because there is nothing to do. The chief interest is to be found in the mediæval architecture. The service of boats between St. Malo and Southampton is only by night. Messrs. Cook have tourist tickets, but they only allow of a short stay and would not, therefore, suit you. Such accommodation as you speak of—quiet lodgings in a village—does not exist in France.

OBER-AMMERGAU AND TIROL (Dolomites).—Miss Davidson's Tour starts on August 18th.

### Notes and Queries.

**REGULATIONS.**

**The Editor is always willing to answer in this column, without any fee, all reasonable questions, as soon as possible.**

**But the following rules must be carefully observed.**

**1. Every communication must be accompanied by the name and address of the writer.**

**2. The question must always bear upon nursing, directly or indirectly.**

**If an answer is required by letter a fee of half-a-crown must be enclosed with the note containing the inquiry.**

*Training.*

(136) Are there any hospitals in England where domestic servants are trained as nurses?—*Nursing Sister.*

Some of the poor-law infirmaries and fever hospitals will accept them.

*Locality.*

(137) I want to know a good locality to start a nursing home in I am a trained nurse, also trained maternity and midwife. What capital would be required?—*M. H.*

The capital required depends largely on the size of the house and the staff kept, but we always try to dissuade nurses from starting nursing homes.

*Castor-oil and Enema.*

(138) 1. Will you kindly inform me the way to give a patient castor-oil in coffee? 2. What kind of gruel is used to make a turpentine enema?—*Nancy.*

1. Pour into a full cup of coffee and let the patient drink the whole dose quickly. 2. Oatmeal gruel.

*Male Nurse.*

(139) I am desirous of becoming a male nurse, but have had no experience and am not able to pay for training. Can you inform me how I might get a start? I am musical, and have had a fair education, am 26 years of age, and stand just upon six feet.—*Bob.*

The Army Medical Corps gives excellent training, or you would be gladly received at one of the mental asylums because of your musical tastes. You might also write to the Matron of the National Hospital for the Paralysed and Epileptic, Queen Square, Bloomsbury, London, W.C.

*Colotomy.*

(140) Can any one give information to a district nurse as to any homes likely to receive a patient who has recently had colotomy performed? She has 8s. weekly.—*M. Stone.*

If the patient resides in London you might write to the Sisters of St. Peter's Home, Mortimer Road, Kilburn, N.W.

*Home.*

(141) You mentioned in THE HOSPITAL the name of a private home at Rickmansworth where a nurse who cannot afford much could stay. Will you kindly give me the address and I shall feel much obliged.—*A. E. A.*

The address is Mrs. Ritchie, Rectory Road, Rickmansworth.

*Children's Hospital.*

(142) I should be grateful if you would tell me where I could find a children's hospital (as near London as possible) where they would take a probationer of 19 or 20. Salary is of no consequence, and I should prefer a place where the nurses are gentlewomen.—*H. O. W.*

East London Hospital, Shadwell, or Evelina Hospital for Sick Children, Southwark Bridge Road, or consult "How to Become a Nurse. The Nursing Profession: How and Where to Train," published by the Scientific Press.

*Consumption.*

(143) I am greatly desirous of ascertaining information as to the whereabouts of consumption sanatoria where they admit necessitous patients either free or at a moderate charge. There was some talk in the papers about building a sanatorium at Davos Platz for poor patients. Has this project been carried out?—*H. A. V.*

You do not state sex of patients. The sanatorium at Davos Platz is not yet built. See "The List of Sanatoria," published by the Society for the Prevention of Tuberculosis, obtainable from the Scientific Press. Price 7d. post free.

**Handbooks for Nurses.**

| | Post Free. |
|---|---|
| "How to Become a Nurse: How and Where to Train." | 2s. 4d. |
| "Nursing: its Theory and Practice." (Lewis.) ... ... | 3s. 6d. |
| "Nurses' Pronouncing Dictionary of Medical Terms." ... | 2s. 0d. |
| "Complete Handbook of Midwifery." (Watson.) ... | 6s. 4d. |
| "Preparation for Operation in Private Houses." ... | 0s. 6d. |

Of all booksellers or of the Scientific Press, Limited, 28 & 29 Southampton Street, Strand, London, W.C.

### For Reading to the Sick.

"THY ROD AND THY STAFF THEY COMFORT ME."

E'EN though thou walkėst through Death's shadowy vale,
Let not thy heart within thee faint nor fail:
His rod shall bud and blossom by the way—
His staff shall comfort in the evil day.

For He is with thee all life's tangled way,
And evil near His presence cannot stay;
By day His sun shall be thy blessėd boon,
And through the night the glory of His moon.

*W. G. K.*

"Thy rod and Thy staff they comfort me." What is this? The rod to correct, the staff to support; the two together forming the Holy Cross. So that the cross of punishment becomes the cross of our support. And we remember in the old history, when the little child was dead, how the prophet sent on his servant to lay his staff on the face of the child. So here, in the solitude and desolation of the soul, when we look up and see that compassionate Form bending over us—"with us" in all the depth of His sympathy, "with us" to catch the fragments of broken prayers which escape our lips, "with us" to cheer and soothe.

"Thou hast been a Strength to the poor, a Strength to the needy in his distress, a Refuge from the storm, a Shadow from the heat, when the blast of the terrible ones is as a storm against the wall." The great Roman, in his magnificent self-confidence, cheered the frightened pilot in the storm, saying, "Fear not; you carry Cæsar." There is One with us, Whom we carry with us, Who can say as no one else can, as wave after wave of sorrow and anguish bursts over our heads, "It is I; be not afraid."—*Canon Newbolt.*

Our blessed Lord passed through the valley of death; we through the valley of the shadow of death. He tasted death that we might never taste of it. He died that we might fall asleep.

PRAYER.

O Lord Jesu Christ, Who by Thy death didst take away the sting of death; grant unto us Thy servants so to follow in faith where Thou hast led the way, that we may at length fall asleep peacefully in Thee, and, awaking up after Thy likeness, may be satisfied with it. *Amen.*

Seek thou the glory of the greater gain
Tread down thy fear, and thou shalt surely see
The meaning of the mystery of pain—
The vision of the Life that is to be:
The summit whence we view the Promised Land—
The splendour of the House not made with hand.

For not in vain thou toilest through the day—
Nor yet in vain doth thy true spirit move
Some other spirit in the toilsome way
The glory of the Eternal Love to prove:
To thee shall come the message of thy Lord—
Love is for evermore Love's sure reward!

## Figures and Tables

**Fig. 3. f1:**